# Cisplatin-Induced Ototoxicity in Head and Neck Cancer: A Systematic Review of Current Evidence, Clinical Implications, and the Need for Standardized Monitoring

**DOI:** 10.3390/curroncol33100584

**Published:** 2026-09-29

**Authors:** Aurora Mirabile, Alert Vukatana, Mathilda Guizzardi, Giorgia Vaccarello, Leone Giordano, Raffaele Addeo

**Affiliations:** 1 Department of Otolaryngology, IRCCS Ospedale San Raffaele, 20132 Milan, Italy; vukatana.alert@hsr.it (A.V.); guizzardi.mathilda@hsr.it (M.G.); vaccarello.giorgia@hsr.it (G.V.); giordano.leone@hsr.it (L.G.); 2 Department of Oncology, P.O. “San Giovanni di Dio”, 50143 Firenze, Italy; raffaele.addeo@aslnapoli2nord.it

**Keywords:** cisplatin, ototoxicity, head and neck cancer, chemoradiotherapy, sensorineural hearing loss, vestibulotoxicity, audiological monitoring, systematic review

## Abstract

**Background:** Cisplatin-based chemoradiotherapy remains a standard treatment for locally advanced head and neck squamous cell carcinoma (HNSCC), but cisplatin-induced ototoxicity is common, usually irreversible and inconsistently monitored. **Methods:** MEDLINE, EMBASE and the Cochrane Library were searched for English-language clinical studies in adults published between January 2016 and January 2026. Primary full-text clinical studies reporting at least one hearing, tinnitus, vestibular or ototoxicity outcome were eligible; conference-only abstracts, duplicate reports, reviews and consensus papers were not. Twenty-four studies were retained from the original database route. A targeted supplementary PubMed verification performed on 16 September 2026 with broader auditory and vestibular terminology identified six additional eligible full-text studies, which were included through the PRISMA 2020 ‘other methods’ pathway, for a total of 30 studies. Risk of bias was appraised for all 30 studies with RoB 2, ROBINS-I and Joanna Briggs Institute (JBI) tools by a single review author; duplicate independent appraisal was not performed and is reported as a limitation. Meta-analysis had been planned but was not undertaken because audiometric frequencies, grading systems, follow-up intervals and effect measures were not comparable; this is reported as a protocol deviation. **Results:** The 30 studies comprised approximately 10,500 participants, although two reports used overlapping Hungarian patient samples and several studies included non-HNSCC populations. Reported ototoxicity ranged from 13% (severe, irreversible loss after weekly cisplatin with cochlear-sparing radiotherapy) to 72% (screen-detected loss in a survivorship clinic), reflecting different definitions rather than a single underlying rate. Comparative HNSCC cohorts consistently reported more hearing loss with 100 mg/m^2^ every three weeks than with weekly 40 mg/m^2^. Ten of the 30 studies were at high, serious or critical risk of bias, with one additional comparative study at moderate-to-serious risk; the long-term randomized comparison reported inferior locoregional control with weekly 30 mg/m^2^. Baseline hearing, cochlear radiation exposure, monitoring adherence and pharmacogenetic susceptibility were further determinants. Objective vestibular dysfunction was documented in the absence of dizziness. No pharmacological otoprotective strategy is established in adults. **Conclusions:** Cisplatin ototoxicity is a core survivorship endpoint. Weekly dosing may reduce hearing toxicity in selected settings but must not be read as universal oncological equivalence. Standardized definitions, baseline and longitudinal testing, cochlear-dose reporting and prospective evaluation of vestibular outcomes and otoprotective agents are the priorities.

## Simple Summary

Cisplatin is an effective part of curative chemoradiotherapy for many head and neck cancers, but it can cause permanent hearing loss and, less commonly recognized, damage to the balance organ. We included 30 clinical studies: 24 unique full-text primary studies retained from the original database search and six additional eligible full-text studies identified by a targeted supplementary PubMed verification on 16 September 2026. Reported figures differ widely, mainly because studies use different tests, definitions and follow-up schedules rather than because the risk itself differs that much. Across comparative head and neck cancer studies, giving cisplatin in lower weekly doses was associated with less hearing damage than giving 100 mg/m^2^ every three weeks; however, the long-term randomized comparison of 30 mg/m^2^ weekly versus 100 mg/m^2^ every three weeks found inferior locoregional control with the lower weekly regimen, so a reduction in hearing damage cannot be equated with equivalent cancer control. Much of the comparative evidence is non-randomized and at serious risk of bias, and hearing tests before, during and after treatment are still performed inconsistently. Standardized auditory and vestibular monitoring is the main unmet need in this field.

## 1. Introduction

Concurrent chemotherapy improves survival when added to radiotherapy in non-metastatic head and neck squamous cell carcinoma (HNSCC), and cisplatin remains the reference radiosensitizer in fit patients [1]. Patient selection matters, because performance status, renal function, comorbidity and pre-existing hearing impairment all modify the risk–benefit balance of platinum [2]. In routine practice cisplatin is given either as 100 mg/m^2^ every three weeks or as a weekly regimen, most often 40 mg/m^2^. These schedules differ in toxicity profile and are not interchangeable across all oncological settings.

**Figure 1:**
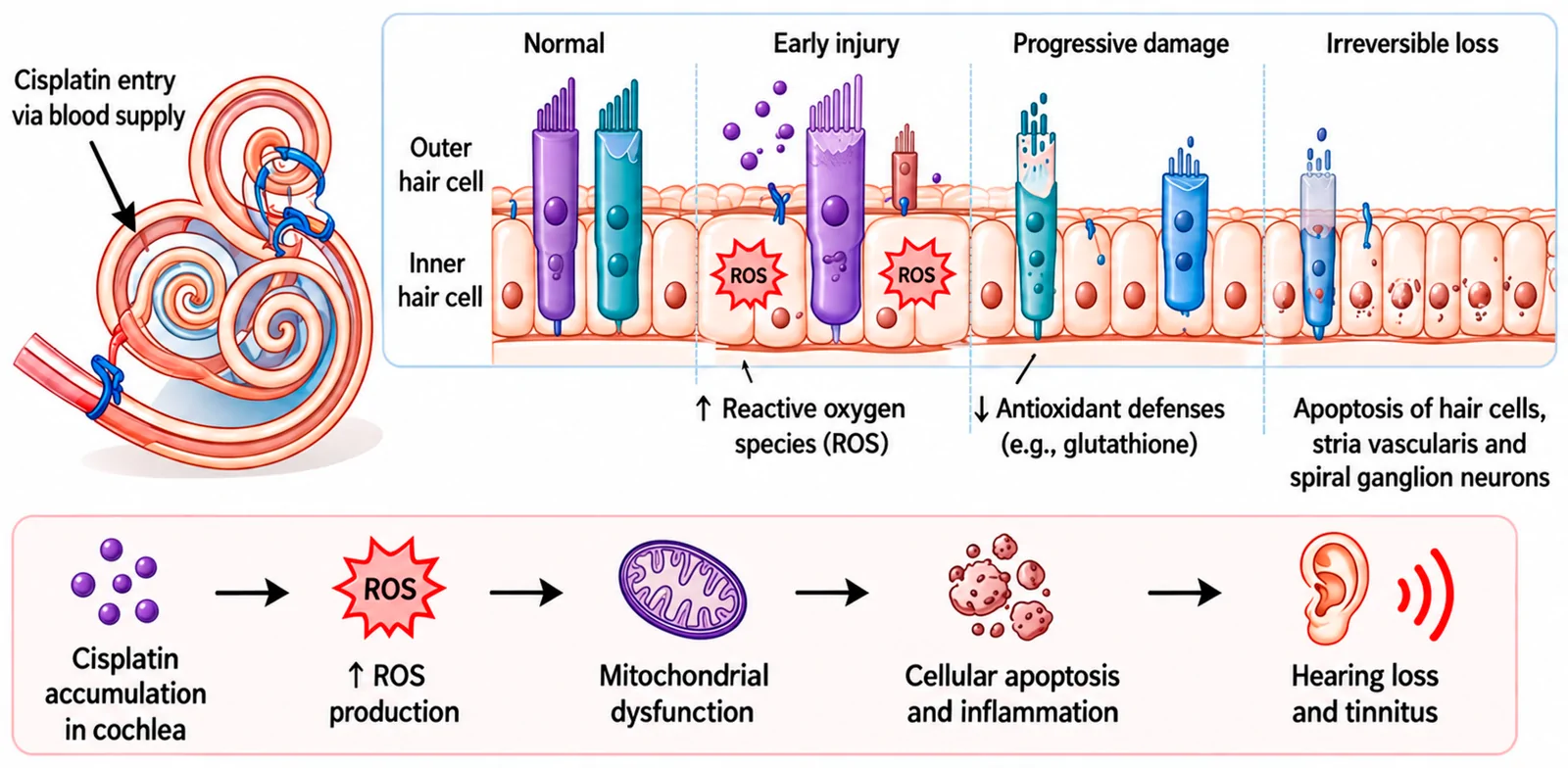
Proposed mechanism of cisplatin-induced ototoxicity. Cisplatin accumulates in the cochlea and promotes oxidative stress, mitochondrial dysfunction, inflammation and apoptosis of cochlear sensory and supporting cells, ultimately resulting in hearing loss and tinnitus.

Cisplatin-induced ototoxicity typically presents as bilateral, predominantly high-frequency sensorineural hearing loss, frequently accompanied by tinnitus. Because the injury is usually permanent and may progress after treatment, it affects communication, social functioning, employment and long-term quality of life [3]. The biological basis is multifactorial and includes reactive oxygen species generation; mitochondrial dysfunction; inflammation and apoptosis of cochlear outer hair cells, the stria vascularis and spiral ganglion neurons (Figure 1).

Head and neck cancer is a particularly complex setting, because systemic cisplatin exposure overlaps with radiotherapy delivered close to the temporal bone. Cochlear dose, cisplatin schedule, cumulative exposure, baseline hearing, renal function, concurrent ototoxic medication and individual susceptibility may all influence the auditory outcome. Monitoring is also inconsistent: audiology guidelines support pre-treatment baseline testing and serial follow-up, yet real-world adherence remains limited [4]. This variability complicates counselling, comparison between institutions and the design of otoprotection trials.

This review synthesizes clinical evidence published between January 2016 and January 2026 around four questions: the reported burden of ototoxicity; treatment- and patient-related risk factors; the evidence for monitoring and prevention; and the methodological standards required of future head and neck cancer studies.

## 2. Materials and Methods

### 2.1. Search Strategy

The systematic search was conducted in MEDLINE, EMBASE and the Cochrane Library for English-language studies published from January 2016 through January 2026; the available search record documents the final search month as January 2026, but the exact calendar day was not preserved. The search strings documented in the original record are reproduced below with parentheses normalized for syntactic clarity; no search concepts were added or removed: “((cisplatin AND ototoxicity) OR (cisplatin AND hearing loss))” and “(((cisplatin AND ototoxicity) OR (cisplatin AND hearing loss)) AND (head and neck cancer))”. Reference lists of eligible reports were inspected for additional candidate studies.

A targeted supplementary PubMed verification was performed on 16 September 2026 using the following query: (cisplatin) AND (ototoxicity OR “hearing loss” OR “sensorineural hearing loss” OR SNHL OR “cochlear damage” OR “cochlear toxicity” OR hypoacusis OR tinnitus OR vestibulotoxicity OR “vestibular dysfunction”) AND (“head and neck cancer” OR HNSCC OR “head and neck neoplasms”). Six additional eligible full-text studies were identified: Teft et al., 2019 [5], Musio et al., 2022 [6], Wheeler et al., 2017 [7], Macedo et al., 2023 [8], Das et al., 2021 [9] and Yadav et al., 2023 [10]. These studies were critically appraised and included through the PRISMA 2020 ‘other methods’ pathway. This verification was not a complete de novo rerun of MEDLINE, EMBASE and the Cochrane Library; the total number of records screened in the targeted verification was not retained, and this limitation is stated explicitly in Section 4.4.

### 2.2. Eligibility Criteria

Eligible studies were primary full-text clinical studies in adults treated with cisplatin-containing chemotherapy or chemoradiotherapy that reported at least one hearing-loss, tinnitus, vestibular or ototoxicity outcome. Randomized trials, prospective and retrospective cohorts, pharmacogenomic studies, radiotherapy-toxicity analyses, monitoring studies and interventional otoprotection studies were eligible. Conference-only abstracts and posters, duplicate or companion reports of an included study, narrative reviews, expert-consensus papers, systematic reviews and meta-analyses, case reports, phase I trials, pilot studies, ongoing studies without results, non-human studies, non-cisplatin regimens, studies in which an auditory outcome could not reasonably be related to cisplatin exposure, and publications outside the predefined window were not eligible.

The registered protocol specified an age range of 18–65 years. During full-text assessment it became clear that this upper limit could not be applied consistently, because the contemporary head and neck cohorts that address the review question routinely enrol patients older than 65 years; the criterion was therefore amended to adults aged 18 years or older, and this amendment is reported as a protocol deviation rather than supported by a post hoc biological rationale. Records excluded at title/abstract screening under the original criterion (n = 59) could not be re-screened within the revision period; they are shown in the flow diagram and the consequence is stated in the Limitations.

### 2.3. Study Selection and Data Extraction

Duplicate records were removed with Zotero (Digital Scholar). Two authors (A.V. and M.G.) screened titles and abstracts, with uncertain records resolved by discussion with a third author (A.M.); retained records were read in full and assessed against the eligibility criteria. Each retrieved full-text record was then re-examined record by record against those criteria, and companion abstracts, duplicate reports, secondary evidence and out-of-window reports were removed from the database-derived included-study count. Extracted variables were study design, setting, population, sample size, cisplatin dose and schedule, radiotherapy exposure and technique, audiological or vestibular assessment method, ototoxicity definition or grading system, follow-up and principal finding. Variables that a study did not report were recorded as not reported and were not imputed. Twenty-four unique full-text primary studies were retained from the database route; the six additional full-text studies identified through the targeted supplementary verification were added via the PRISMA ‘other methods’ route, yielding 30 studies in the final qualitative synthesis. Study-level characteristics are presented in Supplementary Table S1.

### 2.4. Risk of Bias Assessment

Risk of bias was assessed by a single review author using design-appropriate instruments: RoB 2 for randomized trials [11], ROBINS-I for non-randomized comparative studies [12], and the relevant Joanna Briggs Institute (JBI) critical appraisal checklists for single-arm, case–control, cross-sectional and case-series designs [13]. AMSTAR-2 [14] was applied to secondary evidence retrieved at full text but excluded from the primary synthesis. Domain-level judgements for all included studies are reported in Supplementary Table S2, and the results are used in the interpretation of the synthesis (Section 3.2 and Section 4.1). The appraisal was carried out by one reviewer and was not conducted in duplicate by two independent reviewers; this is reported among the limitations in Section 4.4. Certainty of evidence was not formally graded.

### 2.5. Synthesis and Protocol Deviations

The review was conducted and reported in accordance with the PRISMA 2020 statement [15]. The registered protocol anticipated meta-analysis where clinically and methodologically appropriate. After extraction, the audiometric frequencies tested, the ototoxicity definitions and grading systems, the treatment schedules, the follow-up intervals and the reported effect measures were not sufficiently comparable to support a clinically meaningful pooled estimate; the decision not to pool is therefore reported as a deviation from the registered analytic plan. Results are presented as a structured qualitative synthesis, and all numerical estimates quoted are study-level results reported by the original investigators. Two protocol deviations are reported in total: the age criterion (Section 2.2) and the absence of meta-analysis.

### 2.6. Registration

The review was registered in PROSPERO (CRD420261448996).

## 3. Results

### 3.1. Study Selection

**Figure 2:**
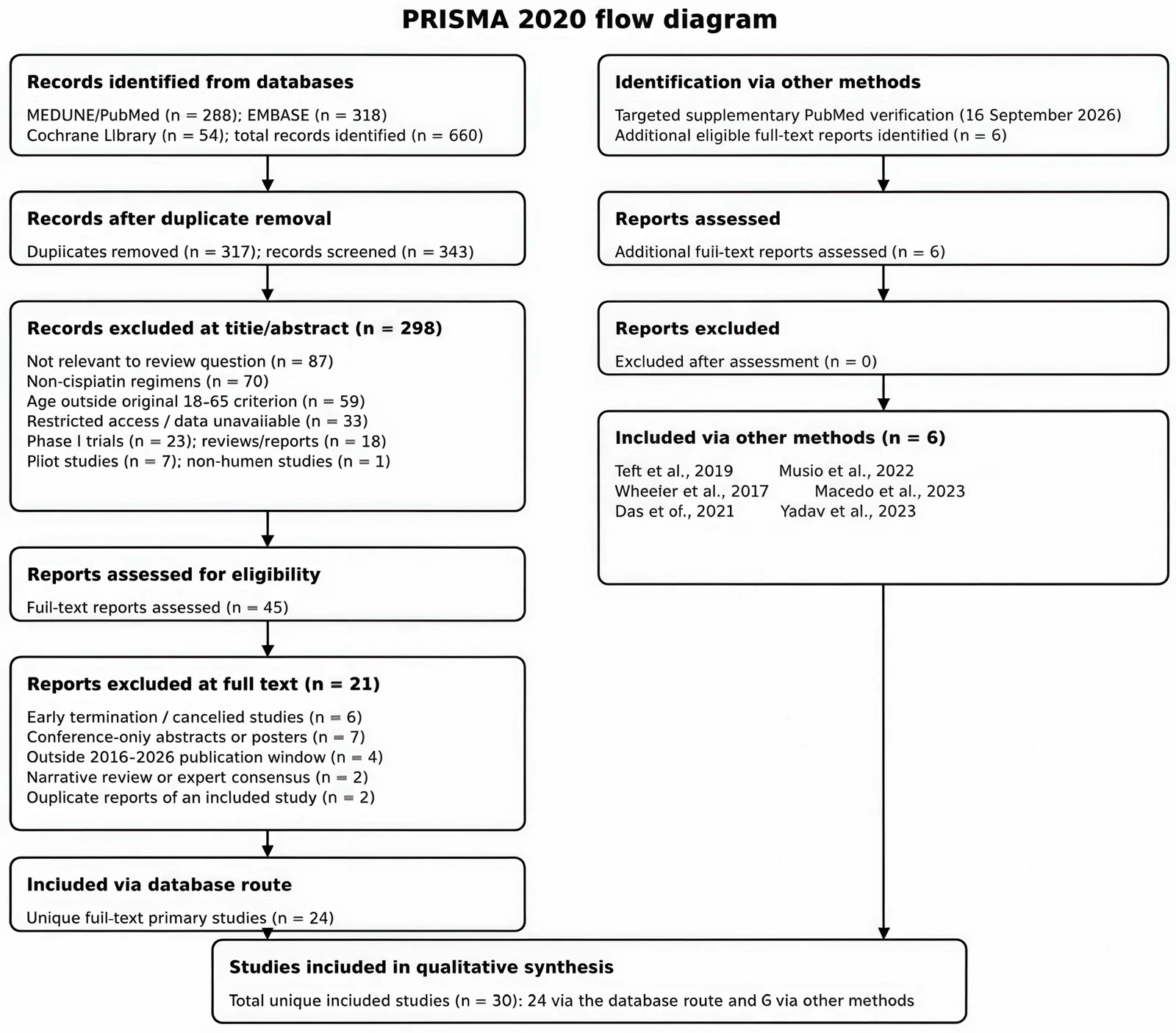
PRISMA 2020 flow diagram of study selection. Twenty-four unique full-text primary studies were retained from the original database route and six additional eligible full-text studies were identified through the targeted supplementary PubMed verification and included via the ‘other methods’ pathway, yielding 30 studies in the final qualitative synthesis. The 59 records excluded under the original age criterion were not individually re-screened. Teft et al., 2019 [5]; Musio et al., 2022 [6]; Wheeler et al., 2017 [7]; Macedo et al., 2023 [8]; Das et al., 2021 [9]; Yadav et al., 2023 [10].

The database search identified 660 records. After removal of 317 duplicates, 343 records were screened by title and abstract and 45 reports were assessed at full text. Twenty-one reports were excluded, with reasons, and 24 unique full-text primary studies were retained from the database route. The targeted supplementary PubMed verification identified six additional eligible full-text studies, all of which were included through the PRISMA ‘other methods’ pathway, yielding 30 studies in the final qualitative synthesis (Figure 2, File S1: PRISMA checklist [15].). Reports excluded at full text from the original database route, with the reason for each, are listed in Supplementary Table S3.

The 30 included studies comprised 7 randomized trials or trial-derived analyses, 11 non-randomized comparative studies and 12 single-arm, cohort, case–control, cross-sectional or case-series studies, and enrolled approximately 10,500 participants in total. The distribution is highly uneven: a single registry cohort contributed 4520 patients [16], a survivorship cohort 1176 [17] and a monitoring audit 379 [18], whereas several audiometrically detailed studies analyzed fewer than 50 patients. Two Hungarian reports [19,20] draw on overlapping patient samples and were counted as separate studies because they address different questions; this is flagged in Supplementary Table S1. Populations ranged from HNSCC-specific cohorts to broader adult cisplatin-treated populations, including testicular and germ cell tumours. Studies identified via the supplementary ‘other methods’ route are marked in Supplementary Table S1.

### 3.2. Risk of Bias

Of the seven randomized studies appraised with RoB 2, two were at high risk of bias, four raised some concerns and one was considered low risk for some concerns; attrition of audiometric data was the dominant problem, with complete audiometric follow-up available in as few as 37% of randomized patients in one trial [21] and 54% in another [22]. Of the 11 non-randomized comparative studies appraised with ROBINS-I, 1 was at critical risk [23], 7 at serious risk [9,24,25,26,27,28,29], 1 at moderate-to-serious risk [30] and 2 at moderate risk [16,31]; the recurrent driver was confounding by indication. Of the 12 studies appraised with JBI checklists, 7 were at moderate risk [5,6,8,10,17,19,32] and 5 at low-to-moderate risk [7,18,20,33,34]. These judgements are carried into the interpretation below: the schedule comparison rests predominantly on non-randomized evidence at serious risk of bias, whereas the randomized evidence is smaller, shorter and affected by missing audiometric data.

### 3.3. Reported Burden of Ototoxicity

No pooled incidence was calculated. Reported estimates spanned an order of magnitude and are not interchangeable, because they measure different things: 13% for severe, irreversible (grade ≥ 3) loss after weekly cisplatin delivered with cochlear-sparing radiotherapy [26]; 18.9% for any-grade hearing dysfunction in the weekly arm and 40.3% in the three-weekly arm of a phase III trial [35]; 19% and 42% for clinically relevant speech-frequency loss after weekly and three-weekly cisplatin respectively [24]; 62% for distortion-product otoacoustic emission (DPOAE) changes after a single cisplatin cycle [19]; and 72% for failure of a screening tone in a survivorship clinic, where only 54% of those failing reported hearing difficulty themselves [17]. Auditory endpoints were captured with *conventional pure-tone audiometry*, *extended high-frequency audiometry*, DPOAEs, Common Terminology Criteria for Adverse Events (CTCAEs) or American Speech-Language-Hearing Association (ASHA) criteria, *patient-reported* hearing handicap and, in one study, *objective vestibular testing. In* one *randomized* trial hearing loss was recorded among adverse events *rather than* as a prespecified auditory endpoint [36], and in one large registry analysis auditory toxicity was captured through coded clinical data rather than audiometry [16]; both are therefore informative about relative frequency rather than about audiometric magnitude.

### 3.4. Cisplatin Schedule and Hearing Outcomes

Comparative studies consistently reported greater hearing toxicity with high-dose three-weekly cisplatin. In a retrospective Dutch cohort of 129 patients, clinically relevant speech-frequency hearing loss occurred in 42% of patients given 100 mg/m^2^ every three weeks versus 19% of those given weekly 40 mg/m^2^ [24]. In 96 patients treated with cochlear-sparing intensity-modulated radiotherapy, grade ≥ 3 irreversible ototoxicity occurred in 56% of the three-weekly group versus 13% of the weekly group (odds ratio 8.4 after adjustment for age, mean cochlear dose and cumulative cisplatin dose) [26]. The multicentre ECHO dataset (n = 564) likewise found lower ASHA- and CTCAE-defined threshold shifts with lower, more frequent dosing [30], and a randomized phase II comparison after induction chemotherapy reported greater loss at 4 and 8 kHz with the higher-dose regimen [21]. Infusion duration and hydration volume did not materially change hearing outcomes within either schedule [25]. In nasopharyngeal carcinoma, cisplatin produced slightly greater early high-frequency loss than carboplatin, a difference that was no longer apparent at 12–18 months [27].

**Figure 3:**
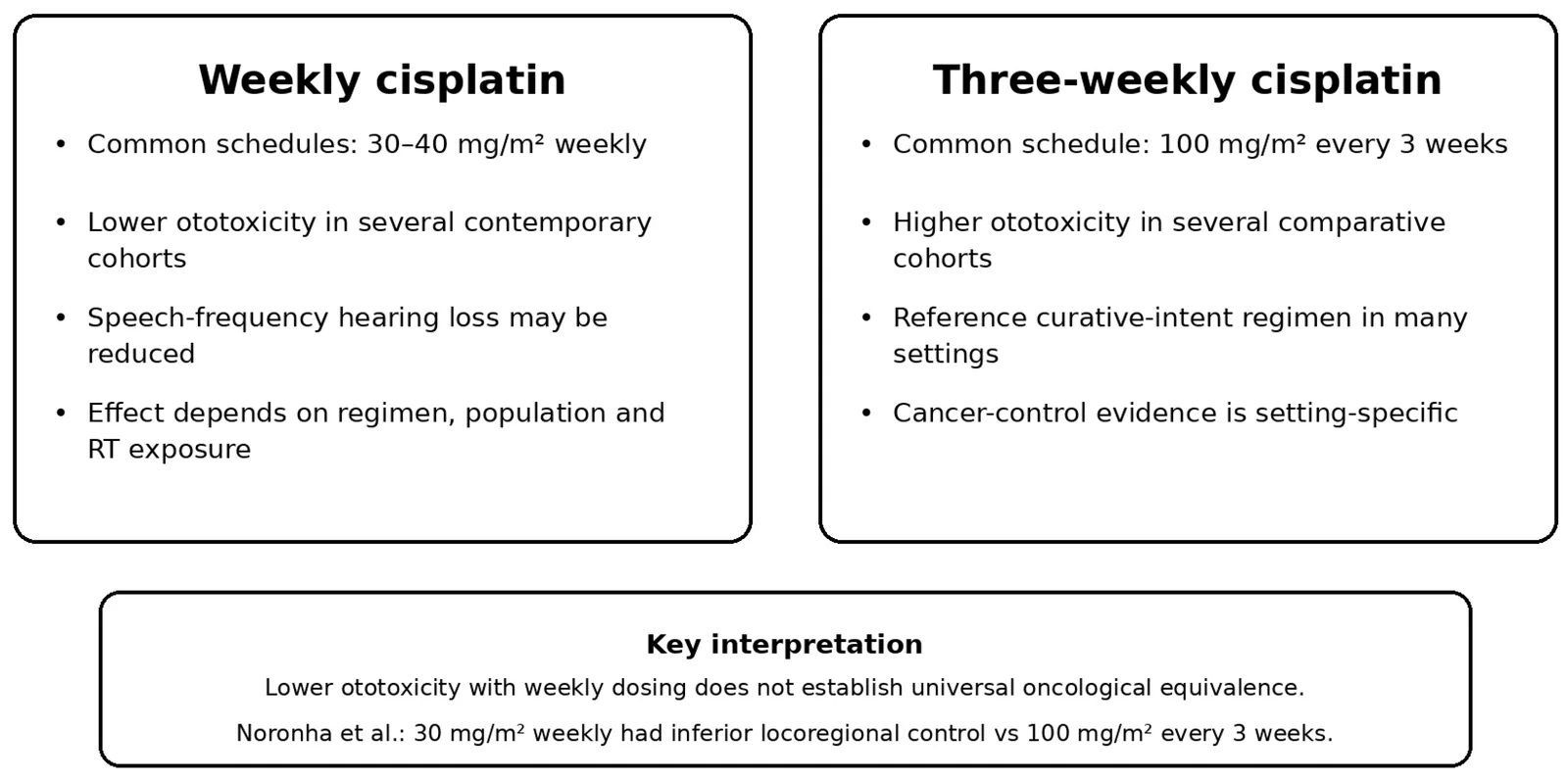
Cisplatin dosing schedule and ototoxicity. Comparative studies generally report a lower ototoxic burden with weekly dosing, but oncological efficacy is regimen- and setting-dependent; reduced hearing toxicity should not be interpreted as proof of universal oncological equivalence [35].

Hearing toxicity must nevertheless be interpreted separately from cancer control. In the long-term update of a phase III randomized trial (n = 300, predominantly adjuvant treatment of oral cavity cancer), cisplatin 30 mg/m^2^ weekly produced less hearing dysfunction but inferior locoregional control compared with 100 mg/m^2^ every three weeks [35]. A retrospective series similarly found inferior survival with weekly doses below 40 mg/m^2^, while ototoxicity was the commonest documented reason for stopping three-weekly treatment [28]. Taken together with the risk-of-bias profile described in Section 3.2, these data indicate a consistent toxicity signal but do not establish oncological equivalence between schedules (Figure 3).

### 3.5. Baseline Hearing, Radiotherapy Exposure and Individual Susceptibility

Baseline hearing status was clinically decisive in the only study designed around it: of 103 patients for whom high-dose cisplatin was planned, 42 (41%) did not start it because of baseline audiometric findings, and 40 of the 61 who were treated (66%) developed marked ototoxicity [23]. Radiotherapy technique also contributes: a prospective cohort treated with low-dose weekly cisplatin and cochlear-sparing intensity-modulated radiotherapy showed only minor audiological change compared with a historical high-dose cohort [29], and severe ototoxicity still occurred in more than half of three-weekly patients despite cochlear sparing [26]. Both studies are at serious risk of bias and neither isolates the radiotherapy contribution, so cochlear-sparing radiotherapy is best regarded as a plausible and low-cost risk-reduction measure rather than as a demonstrated protective intervention.

The targeted supplementary verification added clinically relevant evidence in both HNSCC and non-HNSCC populations. Teft et al. reported a prospective HNSCC cohort with lower ototoxicity under weekly cisplatin and candidate COMT/MATE1 associations [5]. Musio et al. prospectively related cochlear radiation dose to hearing loss in locally advanced head and neck cancer [6]. Wheeler et al. reported an association between WFS1 variation and cisplatin-associated ototoxicity in testicular cancer survivors [7]. Macedo et al. prospectively evaluated clinical and genetic predictors of ototoxicity severity in HNSCC [8]. Das et al. found greater high-frequency sensorineural hearing loss when cisplatin was added to radiotherapy in a prospective head and neck cancer study [9], and Yadav et al. also reported increasing sensorineural hearing loss with higher cochlear radiation dose [10]. Together with the originally included SLC16A5 [33] and GSTM1/GSTT1 [20] studies, these findings support individual susceptibility and cochlear-dose exposure as relevant determinants; the genetic associations remain investigational and should not be used for routine clinical risk stratification.

### 3.6. Audiological Monitoring and Survivorship

Monitoring adherence was poor wherever it was measured. In a cohort of 294 patients treated with cisplatin and radiotherapy, only 74.8% completed at least one post-treatment audiogram, and lack of insurance and stage IV disease predicted complete loss of audiological follow-up [34]. In a 379-patient audit of cisplatin-treated patients, no patient received the complete recommended sequence of baseline, on-treatment and post-treatment audiograms [18]. In a survivorship clinic, 72% of 1176 patients failed screening at one or more frequencies, and only about half of them had reported hearing difficulty [17]. Patient-reported data from a randomized trial showed worse self-perceived hearing handicap after cisplatin-based treatment than after cetuximab-based treatment, sustained to one year [37].

### 3.7. Vestibular Toxicity

Vestibular outcomes were assessed in a single included study. In 45 patients undergoing cisplatin-based chemoradiation, video head impulse testing and vestibular evoked myogenic potentials showed reduced vestibulo-ocular reflex gain and increased corrective saccades six weeks after treatment, in the absence of subjective dizziness and without a significant change in pure-tone audiometry over the same interval [32]. The study is single-arm and cannot separate the cisplatin effect from the concurrent radiotherapy effect, but it indicates that symptom-triggered assessment alone will under-detect vestibular injury.

### 3.8. Otoprotection

**Figure 4:**
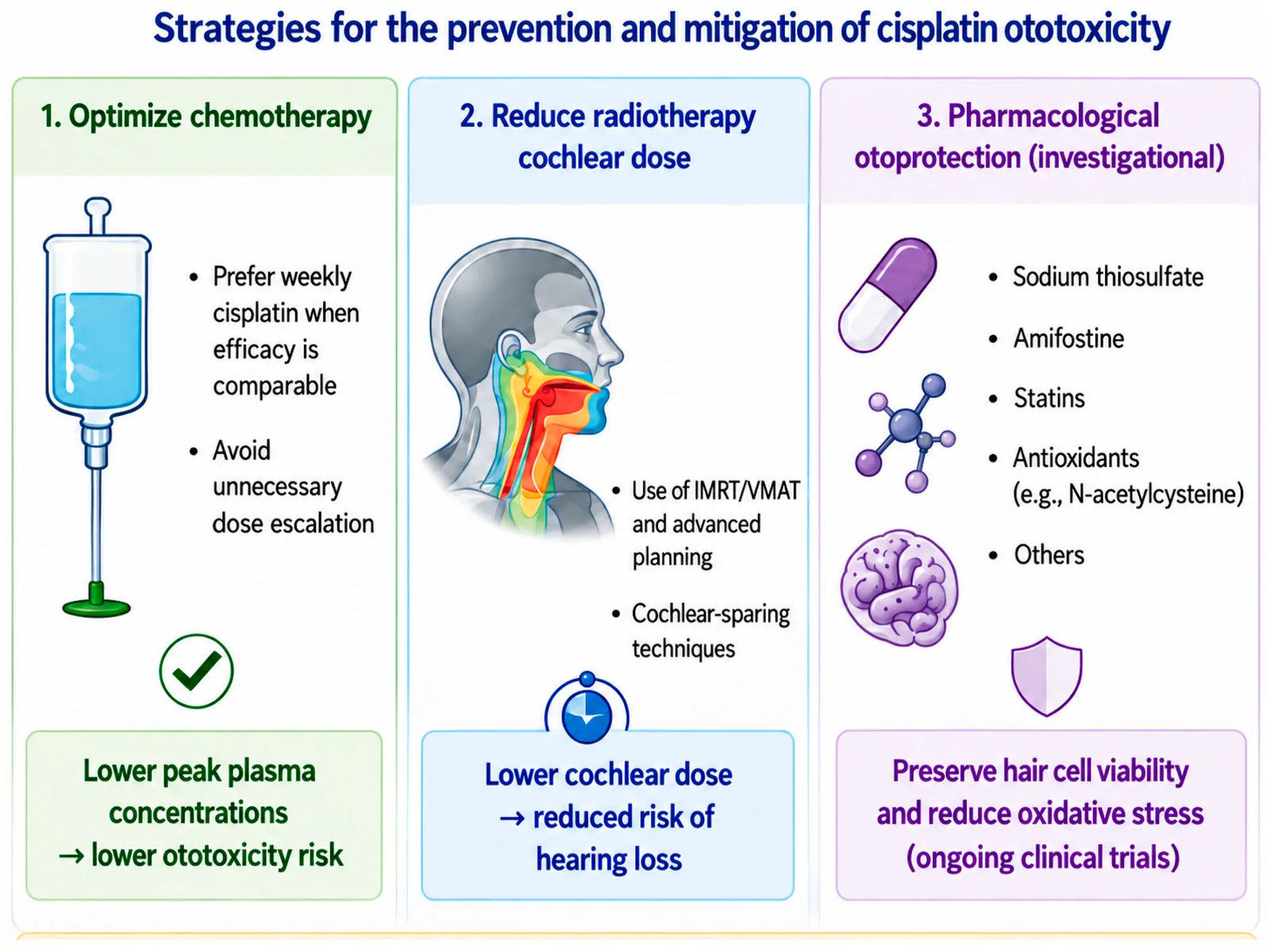
Strategies for the prevention and mitigation of cisplatin ototoxicity. Cisplatin scheduling, cochlear-sparing radiotherapy and pharmacological otoprotection act at different points; pharmacological strategies, including the agents shown, remain investigational in adults and none is established for routine use.

No pharmacological intervention had sufficient randomized evidence in adults with head and neck cancer to support routine use. In an observational cohort of 277 adults, concurrent statin exposure was associated with substantially lower odds of cisplatin-induced hearing loss, although residual confounding cannot be excluded [31]. Low-dose oral N-acetylcysteine did not significantly reduce cisplatin-related toxicities in a randomized head and neck cancer trial [38]. In a phase II randomized trial, D-methionine was associated with an absence of significant threshold shifts at several high frequencies, in contrast with the placebo arm, but only 27 of 50 randomized patients were analyzed and the trial was judged to be at high risk of bias [22]. In a paired-ear phase IIIB trial, intratympanic dexamethasone did not protect hearing and treated ears performed worse than untreated ears [39]. Other systemic otoprotectants, including amifostine and sodium thiosulfate, have been investigated, but adult HNSCC-specific evidence remains insufficient for routine use [3] (Figure 4).

## 4. Discussion

### 4.1. Clinical Interpretation

The most robust conclusion from this evidence set is that cisplatin ototoxicity is multifactorial: per-cycle dose, baseline hearing, cochlear radiation exposure, monitoring strategy and individual susceptibility all contribute. Where weekly cisplatin is an accepted oncological option, the lower ototoxic burden reported across several head and neck cohorts is clinically relevant [24,26,30]. That observation, however, comes overwhelmingly from non-randomized studies at serious risk of confounding by indication, and treatment de-intensification cannot be justified by hearing preservation alone: the long-term randomized comparison shows why toxicity and efficacy must be read together, since weekly cisplatin 30 mg/m^2^ produced less hearing dysfunction but inferior locoregional control [35].

Three practical implications follow. First, baseline audiometry identifies patients in whom the systemic plan should be reconsidered and is the single measure with the clearest supporting evidence [4,23]. Second, the choice of schedule should be made on oncological grounds for the specific setting, with ototoxicity risk as a modifier rather than the determinant. Third, because audiometric thresholds and patient-perceived handicap diverge [17,37], both should be captured before, during and after treatment.

### 4.2. Supplementary-Search and Conference Evidence

The targeted supplementary PubMed verification identified six additional eligible full-text studies that were incorporated through the PRISMA ‘other methods’ route and critically appraised alongside the database-derived studies [5,6,7,8,9,10]. Their direction was concordant with the main synthesis: Teft and Macedo strengthened the HNSCC evidence on clinical and genetic susceptibility; Musio, Das and Yadav supported an association between cochlear radiation exposure and hearing loss; and Wheeler provided the full peer-reviewed WFS1 pharmacogenomic analysis. Their identification also demonstrates that the original search syntax was insufficiently sensitive, which remains a limitation because the full MEDLINE, EMBASE and Cochrane searches were not rerun with the broader strategy.

Conference-only evidence remained outside the synthesis. This included the final and acute-toxicity reports of the randomized RADIO trial comparing weekly and three-weekly cisplatin with audiometric and hearing-related quality-of-life endpoints [40,41]; the direction was concordant with the included comparative cohorts, but the available conference reports did not provide sufficient methodological detail for reliable study-level appraisal. Two conference meta-analyses of cisplatin scheduling were appraised with AMSTAR-2 and rated of critically low confidence because they reported neither a protocol or a reproducible search, nor an appraisal of the pooled trials; they were not used as evidence.

### 4.3. Pharmacological Prevention and Future Trials

Atorvastatin remains the most promising but unproven adult otoprotective strategy, and the observational association reported to date requires randomized confirmation [31]; an ongoing phase II trial sponsored by the National Institute on Deafness and Other Communication Disorders (NIDCD) is evaluating atorvastatin versus placebo in adults with head and neck cancer receiving cisplatin-based chemoradiotherapy [42]. Until randomized efficacy and oncological-safety data are available, pharmacological otoprotection should remain within clinical trials.

**Figure 5:**
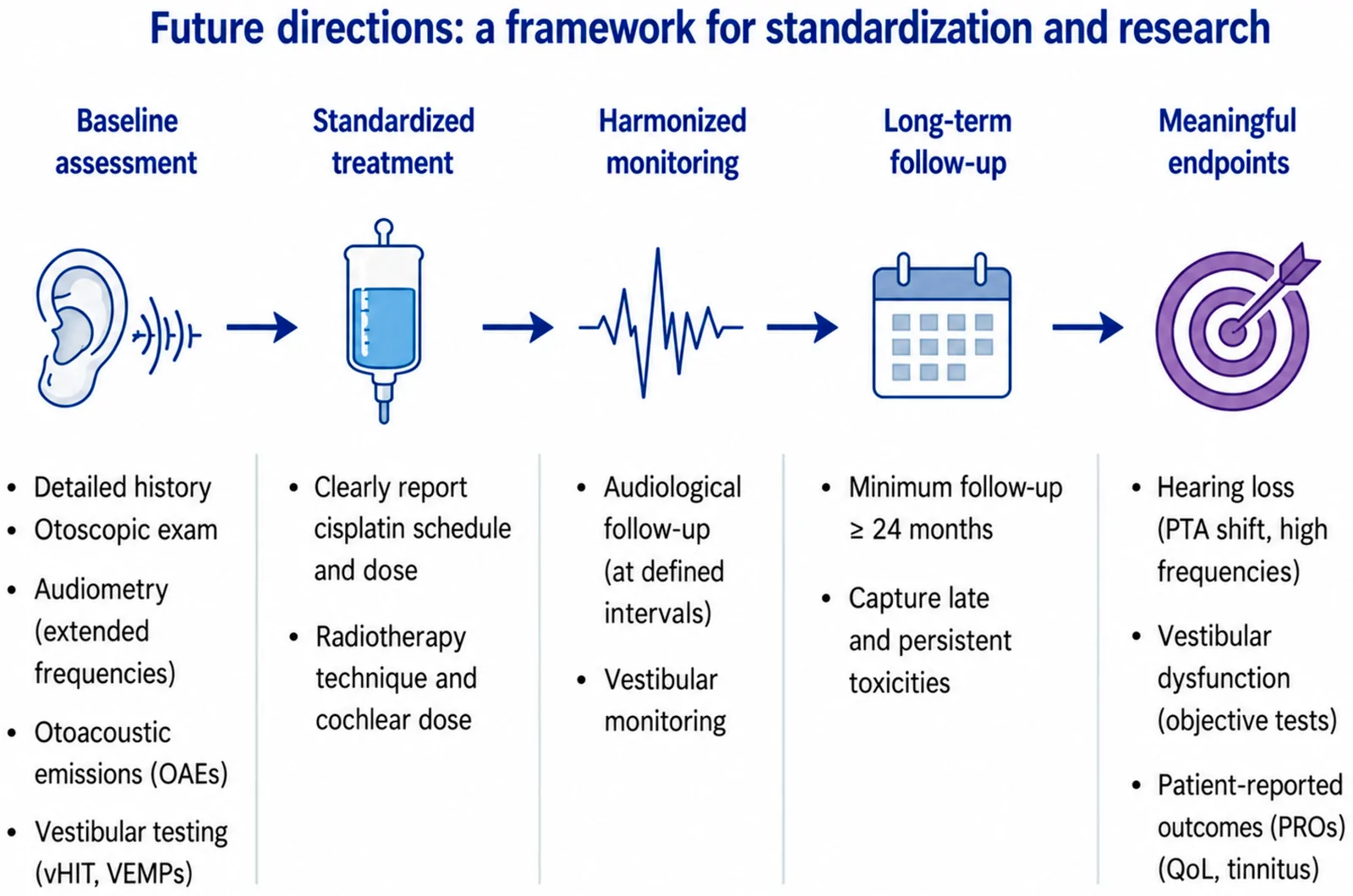
Proposed framework for standardized monitoring and future research, from baseline assessment to harmonized endpoints.

Future head and neck cancer trials involving cisplatin should prospectively specify a minimum ototoxicity dataset: baseline conventional pure-tone audiometry; extended high-frequency audiometry where feasible; prespecified post-treatment time points; one harmonized grading system together with raw threshold shifts; tinnitus and patient-reported hearing outcomes; cumulative and per-cycle cisplatin dose; radiotherapy technique and cochlear dose; documentation of hearing-aid requirement; and vestibular assessment in prospective substudies or in symptomatic patients (Figure 5). Otoprotection trials should be powered for auditory and cancer-control endpoints together.

### 4.4. Limitations

This review has several limitations. First, the original search syntax was narrow: it relied on the terms ototoxicity and hearing loss and did not include several relevant synonyms. A targeted supplementary PubMed verification using broader terminology identified six additional eligible full-text studies and incorporated them through the PRISMA ‘other methods’ route, but the complete MEDLINE, EMBASE and Cochrane searches were not rerun with the broader strategy; moreover, the total number of records screened in that targeted verification was not retained, so residual retrieval bias cannot be excluded. Second, the registered upper age limit of 65 years could not be applied consistently and was amended to adults aged 18 years or older, but the 59 records excluded by age at title/abstract screening were not re-screened, so the review may still under-represent patients older than 65 years. Third, quantitative pooling was not performed because the extracted outcomes were not comparable, so no summary estimate or certainty grading (for example GRADE) is available. Fourth, most of the comparative evidence is non-randomized and at serious risk of confounding by indication, and attrition of audiometric data affected even the randomized trials; in addition, the risk-of-bias appraisal was performed by a single reviewer rather than independently in duplicate, so appraisal error cannot be excluded. Fifth, several mechanistic and pharmacogenomic studies were conducted in testicular or germ cell tumour populations and may not generalize to patients receiving concurrent chemoradiotherapy, and two of them draw on overlapping samples. Sixth, restriction to English-language publications may have excluded relevant studies, and several included studies measured monitoring adherence or screening outcomes rather than audiometrically defined ototoxicity.

## 5. Conclusions

Cisplatin-induced ototoxicity is a frequent and clinically important survivorship problem in head and neck oncology. Contemporary comparative studies consistently report less hearing toxicity with weekly cisplatin schedules than with 100 mg/m^2^ every three weeks, but this evidence is largely non-randomized and at serious risk of bias, and the available long-term randomized comparison shows that a lower weekly dose can compromise locoregional control; reduced ototoxicity must therefore not be equated with oncological equivalence. Cochlear-sparing radiotherapy is a plausible additional measure that does not eliminate systemic cisplatin toxicity, and no pharmacological otoprotective strategy is established for routine adult care. The decisive problem remains methodological: without harmonized audiological definitions, baseline and longitudinal testing, explicit cochlear-dose reporting and prospective evaluation of vestibular outcomes, the field will continue to generate clinically relevant but non-comparable data. Ototoxicity should be treated as a core endpoint of head and neck cancer trials rather than as an optional adverse event.

## Data Availability

No new patient-level data were generated. Study-level data extracted for this review are summarized in Supplementary Tables S1–S3.

## Supplementary Materials

The following supporting information can be downloaded at: https://www.mdpi.com/article/10.3390/curroncol33100584/s1, Table S1, characteristics of the 30 included studies; Table S2, risk-of-bias assessment of the 30 included studies by tool and domain; Table S3, reports excluded at full text from the original database route with reasons; File S1: PRISMA checklist [15].
