# Supplementary material for: Cisplatin-Induced Ototoxicity in Head and Neck Cancer: A Systematic Review of Current Evidence, Clinical Implications, and the Need for Standardized Monitoring
Source: Curr Oncol. 2026 Sep 29;33(10):584. doi: 10.3390/curroncol33100584 (PMC13703248; doi:10.3390/curroncol33100584)
Supplement: Supplementary file 1 [file curroncol-33-00584-s001.zip › curroncol-4505787-supplementary.pdf]

# Supplementary Materials

*Cisplatin-Induced Ototoxicity in Head and Neck Cancer: A Systematic Review of Current Evidence, Clinical Implications, and the Need for Standardized Monitoring*

## Table S1. Characteristics of the 30 included studies

Variables not reported by the original study are marked NR and were not imputed. Reference numbers correspond to the reference list of the main manuscript. Studies marked with an asterisk (*) were identified through the targeted supplementary PubMed verification and included via the PRISMA 2020 "other methods" pathway. CRT, chemoradiotherapy; CTCAE, Common Terminology Criteria for Adverse Events; ASHA, American Speech-Language-Hearing Association; DPOAE, distortion-product otoacoustic emissions; HHIA-S, Hearing Handicap Inventory for Adults–Screening; HPV, human papillomavirus; IMRT, intensity-modulated radiotherapy; NAC, N-acetylcysteine; NR, not reported; q3w, every three weeks; RT, radiotherapy; VEMP, vestibular evoked myogenic potentials; vHIT, video head impulse test; VMAT, volumetric modulated arc therapy.

| Study [ref] | Design and setting | N | Cisplatin exposure / comparison | Radiotherapy technique | Auditory or vestibular assessment and grading | Audiological follow-up | Principal finding | Risk of bias (tool) |
|---|---|---|---|---|---|---|---|---|
| Bauml et al., 2019, Cancer [16] | Retrospective propensity-matched registry cohort; Veterans Health Affairs, USA | 4520 | Cisplatin (n = 3737) vs cetuximab (n = 783) with definitive RT | NR | Toxicity outcomes from coded clinical data, including hearing loss; no audiometric protocol | NR | Cetuximab associated with inferior overall survival; hearing loss captured among recorded toxicities | Moderate (ROBINS-I) |
| Burger et al., 2024, Eur Arch Otorhinolaryngol [24] | Retrospective comparative cohort; single centre, Netherlands | 129 (72 triweekly, 57 weekly) | 100 mg/m² q3w vs 40 mg/m² weekly with CRT | NR | Pure-tone audiometry; clinically relevant hearing loss at 1–2–4 kHz | NR | Speech-frequency hearing loss 42% (triweekly) vs 19% (weekly) | Serious (ROBINS-I) |
| Burger et al., 2025, Oral Oncol [25] | Retrospective cohort; two centres, Netherlands | 161 | Short vs long infusion duration and hydration volume within weekly and triweekly schedules | NR | Pure-tone audiometry; cisplatin-induced hearing loss | NR | No significant difference in hearing loss between infusion/hydration schedules | Serious (ROBINS-I) |
| Caballero et al., 2017, Oncology [23] | Consecutive clinical cohort with audiometry-guided treatment algorithm; Spain | 103 prescribed high-dose cisplatin; 61 treated | High-dose cisplatin 100 mg/m²; switch to a less ototoxic agent if baseline audiometry abnormal | NR | Baseline and serial pure-tone audiometry; decrease > 25 dB as decision threshold | End of treatment | 42/103 (41%) did not start high-dose cisplatin because of baseline audiometry; 40/61 treated (66%) developed marked ototoxicity | Critical (ROBINS-I) |
| Campbell et al., 2022, Int J Audiol [22] | Phase II randomized, double-blind, placebo-controlled trial; India | 50 randomized, 27 analysed | Oral D-methionine vs placebo during cisplatin | NR | Conventional and extended high-frequency pure-tone audiometry | NR | No significant threshold shifts in the D-methionine arm, in contrast with placebo, at several high frequencies | High (RoB 2) |
| Drögemöller et al., 2017, JAMA Oncol [33] | Case-control pharmacogenomic study, discovery and replication; Canada | 188 + 92 (testicular cancer) | SLC16A5 genotype in cisplatin-treated adults | NR | Audiologist-diagnosed cisplatin-induced ototoxicity | NR | SLC16A5 variant carriers less likely to develop ototoxicity; replicated and functionally supported | Low–moderate (JBI, case-control) |
| Driessen et al., 2019, Head Neck [21] | Randomized phase II trial (CONDOR); Netherlands | 62 enrolled; 23 with complete audiometry | Cisplatin 100 mg/m² + conventional RT vs 40 mg/m² weekly + accelerated RT, after induction TPF | Conventional vs accelerated fractionation; technique NR | Serial pure-tone audiometry at 4 and 8 kHz | NR | Greater hearing loss with the high-dose regimen | High (RoB 2); 37% complete audiometric data |
| Fernandez et al., 2021, J Clin Invest [31] | Observational cohort (retrospective with small prospective component); USA | 277 | Concurrent statin vs no statin during cisplatin | NR | Pure-tone audiometry; CTCAE/TUNE grading | NR | Statin exposure associated with ~53% lower odds of cisplatin-induced hearing loss | Moderate (ROBINS-I) |
| Fernandez et al., 2025, JCI Insight [30] | Multicentre cohort (ECHO), five academic centres; USA | 564 | Weekly (< 75 mg/m²) vs triweekly (≥ 75 mg/m²) cisplatin with RT | NR | ASHA and CTCAE v5.0 threshold shift | NR | Lower, more frequent dosing associated with lower incidence and severity of hearing loss | Moderate–serious (ROBINS-I) |
| Gamez et al., 2024, Cancers [26] | Retrospective comparative cohort with prospectively collected audiology; USA | 96 (69 weekly, 27 triweekly) | 40 mg/m² weekly vs 100 mg/m² q3w | Cochlear-sparing IMRT/VMAT in all patients (mean cochlear dose target < 15–20 Gy) | Pure-tone and high-frequency audiometry, DPOAE; CTCAE v5.0 grade ≥ 3 | 3–6 months after CRT | Grade ≥ 3 irreversible ototoxicity 56% (triweekly) vs 13% (weekly); adjusted OR 8.4 | Serious (ROBINS-I) |

| Study [ref] | Design and setting | N | Cisplatin exposure / comparison | Radiotherapy technique | Auditory or vestibular assessment and grading | Audiological follow-up | Principal finding | Risk of bias (tool) |
|---|---|---|---|---|---|---|---|---|
| Hülse et al., 2022, Ear Nose Throat J [32] | Prospective single-arm cohort; Germany | 45 | Cisplatin-based chemoradiation, pre- vs post-treatment | NR | vHIT, cervical and ocular VEMP, pure-tone audiometry | 6 weeks after CRT | Reduced vestibulo-ocular reflex gain and more corrective saccades without subjective dizziness; audiometry unchanged over the same interval | Moderate (JBI, quasi-experimental) |
| Lee et al., 2023, Laryngoscope [34] | Retrospective cohort; single institution, USA | 294 | Cisplatin with radiotherapy | NR | Completion of post-treatment audiograms (monitoring adherence) | Post-treatment, duration NR | Only 74.8% completed ≥ 1 post-treatment audiogram; lack of insurance and stage IV predicted complete loss to follow-up | Low–moderate (JBI, cohort) |
| Lin et al., 2025, Clin Otolaryngol [27] | Retrospective cohort; single centre, China | 86 patients / 156 ears | Cisplatin- vs carboplatin-based induction and concurrent CRT (nasopharyngeal carcinoma) | NR | Bone-conduction threshold shift; CTCAE grade | 12–18 months | Slightly greater early high-frequency loss with cisplatin, not sustained at later follow-up | Serious (ROBINS-I) |
| Moreno & Belinchon, 2022, Ear Hear [39] | Randomized phase IIIB paired-ear trial, masked assessors; Spain | 34 enrolled, 23 analysed (46 ears) | Intratympanic dexamethasone (treated ear) vs untreated contralateral ear during cisplatin | NR | Pure-tone audiometry | NR | No protective effect; treated ears showed worse thresholds than untreated ears | Some concerns (RoB 2) |
| Morse et al., 2019, Head Neck [28] | Retrospective comparative cohort; USA | 163 | Weekly < 40 mg/m² vs weekly 40 mg/m² vs 100 mg/m² q3w with RT | NR | Ototoxicity recorded as documented reason for treatment discontinuation | NR | Inferior survival with weekly doses < 40 mg/m²; ototoxicity the commonest reason for stopping triweekly cisplatin | Serious (ROBINS-I) |
| Niemensivu et al., 2016, Eur Arch Otorhinolaryngol [29] | Prospective cohort with historical comparator; Finland | 22 prospective + 9 historical | Low-dose weekly (prospective) vs high-dose triweekly cisplatin (historical) | IMRT with cochlear sparing | Pure-tone thresholds and tinnitus | NR | Minor audiological change with the weekly, cochlear-sparing approach versus the historical high-dose cohort | Serious (ROBINS-I) |
| Noronha et al., 2025, Int J Radiat Oncol Biol Phys [35] | Phase III randomized trial, long-term update; India (93% adjuvant, oral cavity predominant) | 300 | 30 mg/m² weekly vs 100 mg/m² q3w with RT | NR | Late adverse events including hearing dysfunction (clinical/CTCAE, not audiometry-confirmed) | Long-term update; interval NR | Superior locoregional control with q3w; any-grade hearing dysfunction 40.3% (q3w) vs 18.9% (weekly) | Some concerns (RoB 2) |
| Noszek et al., 2017, Laryngoscope [19] | Prospective single-arm cohort; Hungary | 137 (germ cell tumours) | First cisplatin cycle, pre- vs post-treatment | NR | DPOAE | After the first cycle | 62% showed measurable early ototoxic change after a single cycle | Moderate (JBI, quasi-experimental); overlapping sample with [20] |
| Budai et al., 2020, J Mol Med [20] | Case-control pharmacogenetic study; Hungary | 118 (germ cell tumours) | GSTM1/GSTT1/GSTP1 genotype in cisplatin-treated adults | NR | DPOAE-defined unilateral and bilateral ototoxicity | NR | GSTM1-null predicted unilateral and GSTT1-null bilateral ototoxicity | Low–moderate (JBI, case-control); overlapping sample with [19] |
| Palma et al., 2022, JAMA Oncol (ORATOR2) [36] | Randomized phase II trial; Canada and Australia | 61 | Primary RT with weekly cisplatin vs transoral surgery and neck dissection (HPV-positive oropharynx) | RT, technique NR | Hearing loss captured as adverse event (audiogram-confirmed and self-reported) | NR | Hearing loss uncommon in the cisplatin-containing radiotherapy arm; trial stopped early for surgical-arm toxicity | Some concerns (RoB 2) |
| Runge et al., 2026, Int J Radiat Oncol Biol Phys [37] | Patient-reported outcome substudy of a randomized trial (RTOG 1016); USA/Canada | 375 | RT + cisplatin vs RT + cetuximab (HPV-positive oropharynx) | RT per trial protocol; technique NR | HHIA-S self-perceived hearing handicap | Baseline, end of treatment, 3, 6 and 12 months | Worse self-perceived hearing handicap with cisplatin, sustained to 1 year | Low–some concerns (RoB 2) |
| Santucci et al., 2021, J Otolaryngol Head Neck Surg [18] | Retrospective chart audit; USA | 379 (122 head and neck cancer) | Cisplatin, mixed oncology population | NR | Adherence to recommended baseline, on-treatment and post-treatment audiometry | NR | No patient received the complete recommended monitoring sequence | Low–moderate (JBI, case series) |
| Visacri et al., 2019, Cancer Med [38] | Randomized, double-blind, placebo-controlled trial; Brazil | 57 analysed (28 NAC, 29 placebo) | Oral N-acetylcysteine vs placebo during cisplatin | NR | Ototoxicity among prespecified cisplatin toxicities | NR | Low-dose oral N-acetylcysteine did not significantly reduce cisplatin toxicities, including ototoxicity | Some concerns (RoB 2); 41% attrition |
| Zitelli et al., 2023, J Cancer Surviv [17] | Retrospective survivorship-clinic cohort; USA | 1176 | Head and neck cancer survivorship hearing-screening programme; cisplatin exposure status not specified | NR | Screening at 30 dB HL; amplification uptake | NR | 72% failed screening at ≥ 1 frequency; only 54% of those failing self-reported hearing loss | Moderate (JBI, case series) |
| Teft et al., 2019, Oral Oncology [5]* | Prospective observational HNSCC cohort; Canada | 206 | Cisplatin-based chemoradiotherapy; weekly versus high-dose schedule | Concurrent RT; technique NR | Serial audiometry; CTCAE v4.02 grade ≥2 audiometric change | Baseline, during and after CRT | Weekly dosing associated with less ototoxicity; COMT and MATE1 were candidate susceptibility markers | Moderate (JBI, cohort) |

| Study [ref] | Design and setting | N | Cisplatin exposure / comparison | Radiotherapy technique | Auditory or vestibular assessment and grading | Audiological follow-up | Principal finding | Risk of bias (tool) |
|---|---|---|---|---|---|---|---|---|
| Musio et al., 2022, Anticancer Research [6]* | Prospective single-institution cohort; Italy | 35 | Cisplatin-based CRT for locally advanced HNC | IMRT; cochlear Dmin/Dmean/Dmax evaluated | Serial pure-tone audiometry; study-defined significant hearing loss | Baseline, 1 month, then every 3–6 months | Cochlear radiation dose showed a dose-response association with hearing loss | Moderate (JBI, cohort) |
| Wheeler et al., 2017, Clinical Cancer Research [7]* | Genetic association study; testicular cancer survivors, USA | 511 | Prior cisplatin exposure; WFS1/deafness-gene variants | N/A | Quantitative audiometry at 4–12 kHz; continuous cisplatin-associated ototoxicity phenotype | Long-term survivorship | WFS1 variation was associated with cisplatin-associated ototoxicity | Low–moderate (JBI, analytical cross-sectional/genetic cohort) |
| Macedo et al., 2023, Cancers [8]* | Prospective HNSCC cohort; Brazil | 89 | Cisplatin chemoradiotherapy | Concurrent RT; technique NR | Baseline and post-treatment audiometry plus genotyping | Post-treatment | Clinical and genetic factors were associated with the severity of cisplatin-induced ototoxicity | Moderate (JBI, cohort) |
| Das et al., 2021, Acta Otolaryngologica [9]* | Prospective comparative HNC study; India | 80 | Radiotherapy alone versus concurrent cisplatin chemoradiotherapy | VMAT; cochlear dose assessed | Serial audiometry and otoacoustic emissions; high-frequency SNHL | During and after treatment | Concurrent cisplatin increased high-frequency SNHL relative to radiotherapy alone | Serious (ROBINS-I) |
| Yadav et al., 2023, Indian J Otolaryngol Head Neck Surg [10]* | Prospective HNC cohort; India | 75 | Cisplatin chemoradiotherapy | Various RT techniques; cochlear dose assessed | Pure-tone audiometry; CTCAE / >10 dB SNHL | Baseline, end of treatment and 6 months | Sensorineural hearing loss increased with higher cochlear radiation dose | Moderate (JBI, cohort) |

## Table S2. Risk-of-bias assessment of the 30 included studies by tool and domain

Risk of bias was assessed by a single review author; duplicate independent appraisal was not performed and is reported as a limitation in the main manuscript. Randomized trials and trial-derived analyses were appraised with RoB 2, non-randomized comparative studies with ROBINS-I, and single-arm, cohort, case-control, cross-sectional and case-series designs with the corresponding Joanna Briggs Institute (JBI) checklists. Domain-level judgements are reported below and were used in the interpretation of the synthesis.

### S2a. Randomized studies (RoB 2), n = 7

| Study [ref] | D1 Randomization | D2 Deviations | D3 Missing outcome data | D4 Outcome measurement | D5 Selective reporting | Overall |
|---|---|---|---|---|---|---|
| Campbell et al., 2022 [22] | Low | Low | High (54% retained) | Low | Low | High |
| Driessen et al., 2019 [21] | Low–unclear | Unclear | High (37% complete audiometry) | Low | Some concerns | High |
| Moreno & Belinchon, 2022 [39] | Low | Some concerns (open label) | Some concerns (32% pre-treatment exclusion) | Low | Some concerns | Some concerns |
| Noronha et al., 2025 [35] | Low | Low–some concerns | Some concerns (toxicity denominators < randomized N) | Some concerns (clinical/CTCAE, not audiometry-confirmed) | Low | Some concerns |
| Palma et al., 2022 [36] | Low | Low–moderate (open label, inherent) | Unclear | Low (audiogram) / moderate (self-report) | Low | Some concerns |
| Runge et al., 2026 [37] | Low | Low | Some concerns (attrition across 4 time points undetailed) | Low (validated instrument) | Low | Low–some concerns |
| Visacri et al., 2019 [38] | Low | Low | Serious (97→57 analysed, 41% loss) | Low | Low | Some concerns |

### S2b. Non-randomized comparative studies (ROBINS-I), n = 11

| Study [ref] | D1 Confounding | D2 Selection | D3 Classification | D4 Deviations | D5 Missing data | D6 Outcome measurement | D7 Selective reporting | Overall |
|---|---|---|---|---|---|---|---|---|
| Bauml et al., 2019 [16] | Moderate | Low | Low | Unclear | Low–moderate | Low | Low | Moderate |
| Burger et al., 2024 [24] | Serious | Unclear | Low | Unclear | Unclear | Low | Low | Serious |
| Burger et al., 2025 [25] | Serious | Low–moderate | Low | Unclear | Unclear | Low | Low | Serious |
| Caballero et al., 2017 [23] | Critical | Unclear | Low–moderate | Unclear | Unclear | Low | Unclear | Critical |
| Fernandez et al., 2021 [31] | Moderate | Low–moderate | Low | Low–unclear | Unclear | Low | Low | Moderate |
| Fernandez et al., 2025 [30] | Moderate–serious | Moderate | Low | Low | Low–unclear | Low | Low | Moderate–serious |
| Gamez et al., 2024 [26] | Serious | Low–moderate | Low | Moderate | Low–moderate | Low | Low | Serious |
| Lin et al., 2025 [27] | Moderate | Low–moderate | Low | Unclear | Serious | Low | Low–moderate | Serious |
| Morse et al., 2019 [28] | Serious | Low–moderate | Low | Moderate | Unclear | Serious | Low–moderate | Serious |
| Niemensivu et al., 2016 [29] | Serious | Moderate | Low | Unclear | Unclear | Moderate | Low | Serious |
| Das et al., 2021 [9] | Serious | Moderate | Low | Unclear | Low–moderate | Low | Low | Serious |

**S2c. Single-arm, cohort, case-control, cross-sectional and case-series studies (JBI), n = 12**

| Study [ref] | JBI checklist used | Key strengths | Key weaknesses | Overall |
|---|---|---|---|---|
| Drögemöller et al., 2017 [33] | Case control | Independent replication cohort and functional assays; standardized dual-audiologist outcome | Cases and controls not matched for age, dose or number of cycles (adjusted analysis only); testicular cancer population | Low–moderate |
| Hülse et al., 2022 [32] | Quasi-experimental (single arm) | Standardized vHIT, VEMP and pure-tone audiometry at three time points | No comparator group; cisplatin effect cannot be separated from concurrent radiotherapy | Moderate |
| Lee et al., 2023 [34] | Cohort | Multivariable-adjusted predictors; standardized time points | Measures monitoring adherence rather than ototoxicity incidence; audiograms obtained outside the system cannot be excluded | Low–moderate |
| Noszek et al., 2017 [19] | Quasi-experimental (single arm) | Objective DPOAE outcome; paired analysis | No comparator; single pre/post time point (short-term only); germ cell tumour population; overlaps [20] | Moderate |
| Budai et al., 2020 [20] | Case control | Objective DPOAE outcome; multivariate adjustment | No replication cohort; germ cell tumour population; overlaps [19] | Low–moderate |
| Santucci et al., 2021 [18] | Case series | Large chart audit with clear inclusion criteria | Chart-based ascertainment probably undercounts; head and neck cancer only 32% of the cohort; measures adherence rather than incidence | Low–moderate |
| Zitelli et al., 2023 [17] | Case series | Large sample; standardized screening protocol | Coarse pass/fail screening tone rather than full audiometry; cisplatin exposure status unspecified | Moderate |
| Teft et al., 2019 [5] | Cohort | Prospective HNSCC cohort; standardized serial audiometry; multivariable analysis | Non-randomized schedule allocation; residual confounding by indication | Moderate |
| Musio et al., 2022 [6] | Cohort | Prospective cochlear dosimetry and serial audiometry | Small single-centre cohort; no external validation; residual treatment confounding possible | Moderate |
| Wheeler et al., 2017 [7] | Analytical cross-sectional / genetic cohort | Large sample; objective multi-frequency audiometry; genetic association supported by functional analyses | Survivor cohort; non-HNSCC population; residual phenotype heterogeneity | Low–moderate |
| Macedo et al., 2023 [8] | Cohort | Prospective HNSCC cohort; baseline/post-treatment audiometry; genetic analysis | Single-centre study; multiple candidate variants; limited external validation | Moderate |
| Yadav et al., 2023 [10] | Cohort | Prospective audiometry with cochlear-dose assessment | Single-centre cohort; treatment heterogeneity; limited multivariable confounder control | Moderate |

Summary: of the 7 randomized studies, 2 were at high risk of bias, 4 raised some concerns and 1 was at low risk to some concerns. Of the 11 non-randomized comparative studies, 1 was at critical risk, 7 at serious risk, 1 at moderate-to-serious risk and 2 at moderate risk. Of the 12 studies appraised with JBI checklists, 7 were at moderate and 5 at low-to-moderate risk. The two conference meta-analyses retrieved at full text were appraised with AMSTAR-2 and rated of critically low confidence; they were not used as evidence.

## Table S3. Reports excluded at full text from the original database route with reasons

Twenty-one reports were excluded at full text from the original database route. Twelve are listed individually below. The remaining nine were excluded during the original screening process as early-terminated or cancelled studies (n = 6) and as reports outside the predefined publication window (n = 3).

| Report | Reason for exclusion | Note |
| --- | --- | --- |
| Ahn et al., 2016, Oral Oncology | Expert consensus and narrative review | Not primary clinical research; retained as a contextual citation only |
| Chattaraj et al., 2023, JCO Oncology Practice | Narrative review with practice-pattern survey | Publication type is review; measures practice patterns, not ototoxicity incidence; retained as a contextual citation |
| Driessen et al., 2016, Ann Oncol (ESMO abstract 989P) | Conference abstract; companion report | Interim report of the trial published in full and included as Driessen et al. 2019 |
| Gul et al., J Clin Oncol (abstract e18063) | Conference abstract; secondary evidence (meta-analysis) | AMSTAR-2 confidence critically low: no protocol, no reproducible search, no appraisal of pooled trials |
| Gul et al., J Clin Oncol (abstract e18063) — second copy | Duplicate report | Duplicate of the record above |
| Gul et al., J Clin Oncol (abstract e18121) | Conference abstract; secondary evidence (meta-analysis) | AMSTAR-2 confidence critically low |
| Hülse et al., 2022 — second copy | Duplicate report | Duplicate of the included study [32] |
| Kuruvilla et al., 2024, Ann Oncol (LBA37, RADIO trial) | Conference abstract | Randomized trial with audiometric co-primary endpoint; discussed in Section 4.2 as evidence not included in the synthesis |
| Winquist et al., 2024, Int J Radiat Oncol Biol Phys (RADIO trial, acute toxicity) | Conference abstract; companion report | Companion acute-toxicity report of the trial above; discussed in Section 4.2 |
| Page et al., Int J Radiat Oncol Biol Phys (ASTRO abstract) | Conference abstract | Feasibility study of portable audiometry (n = 32); no full publication retrieved |
| Wheeler et al., 2016, J Clin Oncol (ASCO abstract) | Conference abstract | Superseded by the full publication (Wheeler et al., 2017), identified by the supplementary verification and included via the PRISMA “other methods” pathway as study [7] |
| Nagy et al., 1999, Am J Clin Oncol | Outside the 2016–2026 publication window | Mixed head and neck, oesophageal and lung cancer population |
